# Editorial: Root Branching: From Lateral Root Primordium Initiation and Morphogenesis to Function

**DOI:** 10.3389/fpls.2019.01462

**Published:** 2019-11-18

**Authors:** Joseph G. Dubrovsky, Hidehiro Fukaki, Laurent Laplaze, Marta Laskowski

**Affiliations:** ^1^Departamento de Biología Molecular de Plantas, Instituto de Biotecnología, Universidad Nacional Autónoma de México, Cuernavaca, Mexico; ^2^Department of Biology, Graduate School of Science, Kobe University, Kobe, Japan; ^3^DIADE, Université de Montpellier, IRD, Montpellier, France; ^4^Biology Department, Oberlin College, Oberlin, OH, United States

**Keywords:** plant development, root system architecture, lateral root, pericycle, branching, morphogenesis

## Why Study Root Branching?

Out of the total biomass of all life kingdoms on our planet (550 Gigatons of C; 1Gt C = 10^15^ g of carbon), belowground biomass, most of which is composed by plant roots, is estimated to be 130 Gt of C (Bar-On et al., 2018). Roots are important not only for soil formation, maintenance, and C input but also for the whole of plant life as they provide and transport water and minerals to the above-ground organs that permit successful photosynthetic nutrition. To forage for nutrients and water, roots grow and spread widely throughout the soil. Their spread is promoted by extensive branching; one plant may have millions of lateral roots (LRs). Despite the importance of LRs, many questions regarding their initiation and development are still not understood. Increasing our understanding of the mechanisms shaping root system architecture (RSA) has become an essential component in devising new strategies to cultivate and breed plants that are more resilient to abiotic stresses, a factor whose importance is rising as the growing human population demands sustainable intensification of agricultural practice and as the impact of global changes increases.

## Lateral Root Initiation and Competent State of the Pericycle

The first steps in LR formation are related to priming and commitment of the pericycle cells in which the first divisions leading to LR primordium (LRP) formation occur. To elucidate the molecular events that take place during LR initiation and primordium development at cellular/tissue levels, cell type-specific transcriptomics of LR formation based on fluorescent activated cell sorting or laser capture microdissection has been performed using *Arabidopsis thaliana* and cereal model systems, thereby providing us the useful gene catalogues of genes involved in LR initiation. Kortz et al. review the recent advances obtained using this approach and focus on cell type-specific responses to nitrate-linked LR formation in maize, in which auxin transport through ZmPINs and cell cycle inhibition by Kip-related proteins are involved in LR branching from shoot-born brace roots in maize.

Participation of different hormones, chiefly auxin, in LR initiation has been extensively studied in *A. thaliana* and is reviewed by Torres-Martínez et al. In *A. thaliana*, auxin can both promote and inhibit LR formation, depending on its concentration (Ivanchenko et al., 2010). Importantly, Alarcón et al. found a similar tendency in a monocot species, *Zea mays*. They show that relatively high concentrations of exogenous auxin inhibit LR formation in just that part of the root formed after hormone treatment, and this reduction is accompanied with a reduced pericycle cell length. Their data suggest that pericycle cells undergo a period of competence after which LR initiation does not take place and that root growth, pericycle cell length and LR formation are linked and can be regulated by auxin.

In squash, *Cucurbita pepo*, LR founder cells divide within the root apical meristem rather than the young differentiation zone as occurs in *Arabidopsis*. Kiryushkin et al. used phylogenetic analysis together with auxin-responsive expression in the root to search for putative functional orthologs of two *Arabidopsis* genes associated with the early stages of LR development: *GATA23* and *MAKR4*. They showed that expression of both genes starts in the protoxylem and then spreads to the pericycle founder cells. The authors find it unlikely that there is enough space for auxin oscillations to lead to formation of pre-branch sites prior to *CpMAKR4* expression. In addition, it appears that LR initiation in squash is not induced by an inward-moving auxin signal such as might arise from dying root cap cells.

In the lycophyte, *Selaginella moellendorffii*, roots arise from stem-born structures called rhizophores. Within each root, a single, tetrahedrally-shaped stem cell (apical cell) nucleates the production of new cells that permit growth. These roots branch dichotomously when the root tip bifurcates, with each side having its own apical cell. Fang et al. investigated the extent to which auxin serves as a signal for the formation of new roots in *Selaginella* and concluded that while the plant responds to auxin, root production is indirectly affected, suggesting that the new apical cells formed during root tip dichotomous branching may be generated by an auxin-independent mechanism.

## Morphogenesis of Lateral Root Primordia

In contemporary studies and reviews, the literature considered is commonly not older than two-three decades. Torres-Martínez et al. made an effort to integrate our knowledge of LR morphogenesis in both old and new literature embracing all angiosperms that include >15,000 genera (Soltis et al., 2018). They overview the participation of pericycle cells and other tissues in LRP morphogenesis and attempt to outline the phylogeny of a temporary cap-like structure of endodermal origin in LRP morphogenesis. Also, they identify categories of mutants affected in LRP morphogenesis and address genetic control and the roles of mechanical forces, cell proliferation, patterning, and cell identity acquisition in LRP morphogenesis.

## Root Branching Plasticity, Nutrients and Modeling

Compared to the eudicot plants such as *A. thaliana*, monocots such as maize plants form a structurally and functionally complex root system consisting of different root types (primary, seminal, lateral, and crown roots), which shows root branching plasticity. Yu et al. provide an update on the molecular mechanisms in the LR branching response to environmental signals such as nutrients and water in maize. The authors explain the architectural responses of maize LRs to the availability of soil resources such as nitrate, phosphate and water. The LR branching response to the uneven distribution of water and nutrients in soil is also discussed with the aid of molecular genetic data obtained in *A. thaliana* and maize.

Root branching is a plastic trait that responds to signals from other parts of the plant. For instance, root tip excision promotes the developmental progression of previously specified lateral root primordia. Justamante et al. describe the natural variation in RSA after root tip excision among 120 A*. thaliana* accessions. Using a genome-wide association study, they identified 19 genomic loci involved in the regulation of excision-induced changes in RSA. Three candidate loci associated with wound-induced LR formation were further investigated and a potential mechanism involving cytokinin is proposed.

In addition to cereals, root and tuber crops (RTCs) such as cassava, potato, sweet potato, and yams, are also important as a global source of carbohydrates, particularly in regions not suitable for cereal production. However, root branching traits that enhance nutrient acquisition in RTCs has not been well studied. Duque and Villordon provide a comprehensive review of recent literatures on RTCs, including the authors’ research on RSA traits under phosphorus deficiency in sweet potato. To manipulate RSA for increased nutrient efficiency in RTCs, new research directions are proposed including strategic translational research using molecular genetic data on RSA and nutrient uptake from *A. thaliana* and cereal model systems.

Root branching is an important component of RSA and has therefore an important role in the adaptation of different plant species to their specific ecosystem. The article by Pages describes an analysis of the inter- and intraspecific variations in inter-branch distance in 36 plant species collected in homogenous soil conditions. It proposes a simple model based on three parameters accounting for two processes: the location of potential branching sites along the root and the emergence of LRs at these sites that simulates the observed variations in the different species. This suggests that these parameters could be useful traits for analysis of plasticity in root branching dependent on genotype and environmental conditions.

## Future Perspectives

To understand the evolution of root system development and branching as well as the biology of crop species, research programs gradually move from a model species, *A. thaliana*, to a wider spectrum of other angiosperms and vascular plants in general. Clearly, this tendency will be maintained. Development of new methodological approaches such as single cell transcriptomics, 4-D imaging microscopy, new phenomics systems, including computer tomography, modeling at different organization levels, and other innovations are already starting and will help further our understanding of root branching mechanisms. It is a challenge for our community to integrate all this information and comprehend root branching, from LRP initiation and morphogenesis to evolution and function.

## Author Contributions

JD prepared the outline of the manuscript. All authors wrote parts of the manuscript, improved the draft and revised the final version.

## Funding

The work in the JD laboratory is partially supported by DGAPA-PAPIIT-UNAM grant IN200818 and CONACyT grant A1-S-9236. The work in the HF laboratory is supported by Grant-in-Aid for Scientific Research (B)(18H02463) from the MEXT, Japan. Work in the ML laboratory is supported by NSF IOS – 1656621, USA. LL acknowledges support from the French National Research Agency (ANR) through the NewRoot project (ANR-17-CE13-0004-01).

## Conflict of Interest

The authors declare that the research was conducted in the absence of any commercial or financial relationships that could be construed as a potential conflict of interest.
